# Supplementary material for: Content-rich biological network constructed by mining PubMed abstracts
Source: BMC Bioinformatics. 2004 Oct 8;5:147. doi: 10.1186/1471-2105-5-147 (PMC528731; doi:10.1186/1471-2105-5-147)
Supplement: Additional File 5 — The original Chilibot query results of the term "long-term potentiation (LTP)" and 22 other terms, limiting the latest references analyzed to the years 1990, 1995, 2000, and 2004. [file 1471-2105-5-147-S5.bz2 › chilibotAdditionalFile5/ltp2004/html/SYNAPSIN I_SYNAPTOTAGMIN.html]

 


 **SYNAPSIN I** and **SYNAPTOTAGMIN** 
  
Found 25 abstracts in PubMed,  **25 abstracts were retrieved and analyzed**.  


---

 Search Google  |
 PDF files only 
|  EDU domain only 

---

**Interactive relationship** (e.g. stimulation, inhibition, etc)

**Parallel relationship** (e.g. studied together, co-existance, homology, etc.)

- Our results show that the levels of  **synapsin I**  and  **synaptotagmin**  I are increased in the hypothyroid rats, which suggests that the genes encoding these proteins are implicated in the action of thyroid hormone on neurotransmitter release.Taken together, the results from this study suggest that thyroid hormone may modulate the probability of neurotransmitter release.  Ref: 11882369 Neuroscience, 2002
- To investigate the changes induced in presynaptic protein phosphorylation by 5 HT reuptake blockade and concomitant CaMKII up regulation, we analyzed two major CaMKII presynaptic substrates  **synapsin I**  and  **synaptotagmin** .  Ref: 9016342 Mol Pharmacol, 1997
- The modulation of ischemic hippocampal damage by the in vivo knockdown of  **synaptotagmin**  I or  **synapsin I**  suggests that transmitter release from terminals plays an important role in the evolution of ischemic brain damage and therefore the transmitter release strategy by the use of antisense ODNs HVJ liposome complex is reliable for neuroprotective therapies.  Ref: 12631464 Neurosci Res, 2003
- Horseradish peroxidase filled vacuoles as wells as vesicles contained the synaptic vesicle membrane proteins VAMP II,  **synaptotagmin** , SV2, and synaptophysin, the vesicle associated proteins rab 3A and  **synapsin I** , and in addition SNAP 25.  Ref: 10579591 Neuroscience, 1999
- The antisense in vivo knockdown of  **synaptotagmin**  I prevented almost completely the ischemic damage of hippocampal CA1 neurons, while the in vivo knockdown of  **synapsin I**  markedly promoted the ischemic damage of CA1 pyramidal neurons and extended the injury to relatively resistant CA2 CA3 region.  Ref: 12631464 Neurosci Res, 2003
- Despite the nearly constant rates of synthesis of  **synaptotagmin**  I, vamp 2, and  **synapsin I** , we show that the number of SVs in these developing neurons increases,.  Ref: 9065497 J Neurosci, 1997
- The conditions used indicate that  **synaptotagmin** , as  **synapsin I** , is phosphorylated by calcium calmodulin dependent protein kinase II.  Ref: 8381369 FEBS Lett, 1993
- Rab3a and synaptobrevin II were retained at a high level and  **synaptotagmin**  I,  **synapsin I** , and syntaxin I at a lower level until 20 DIV.  Ref: 10340764 Glia, 1999
- High magnification revealed that  **synaptotagmin**  I like immunoreactivity was mainly distributed in a fine granular pattern, but large, brightly fluorescent granules which were not labelled by anti synaptophysin or anti  **synapsin I**  were occasionally observed.  Ref: 7534885 Neuroscience, 1994
- No  **synaptotagmin**  I like immunoreactivity was found in large axons, while accumulations of the synaptic vesicle proteins synaptophysin and  **synapsin I**  were found in all types of axons.  Ref: 7534885 Neuroscience, 1994
- In parallel, there was a substantial increase in the expression levels of the presynaptic markers synaptophysin,  **synaptotagmin** , and  **synapsin I**  and of GluR1 AMPA receptor subunits.  Ref: 12595243 Mol Cell Neurosci, 2003
- We measured the levels of seven presynaptic proteins synaptobrevin,  **synaptotagmin** , SNAP 25, syntaxin, SV2, Rab3a, and  **synapsin I**  by immunoblotting in postmortem tissue from four brain regions hippocampus, entorhinal cortex, caudate nucleus, and occipital cortex.  Ref: 10831767 J Neurol Sci, 2000
- At both 4 and 12 DIC, the synaptic proteins rab3a,  **synapsin I** , and  **synaptotagmin**  were present in hippocampal neurons, but the subcellular distribution changed from one in which immunoreactivity was initially distributed within soma and neurites to a punctate varicose appearance.  Ref: 7965045 J Neurosci, 1994
- The amount of immunoreactive  **synapsin I** , synaptophysin, and  **synaptotagmin**  was not appreciably changed for 72 hours after reperfusion.  Ref: 11988617 Stroke, 2002
- In order to examine the possible molecular mechanisms underlying these synaptic changes, we compared the expression of  **synapsin I** ,  **synaptotagmin**  I, syntaxin, and alpha calcium calmodulin kinase II between control and hypothyroid hippocampus.  Ref: 11882369 Neuroscience, 2002
- In order to investigate the effects of antidepressant compounds on SVP mRNA levels, the expressions of synaptophysin,  **synaptotagmin** , VAMP, and  **synapsin I**  were analysed by in situ hybridization in rats which had been treated with desipramine, fluoxetine, tranylcypromine, or saline.  Ref: 14708000 Pharmacogenomics JPharmacogenomics J, 2002
- VAChT containing varicosities in the Renshaw cell area, with a probable origin from motoneuron axon collaterals, exhibited CSP,  **synapsin I**  II, and synaptobrevin 1 LI... syntaxin, synuclein,  **synapsin I** ,  **synaptotagmin**  I,  **synaptotagmin**  I II, synaptophysin and synaptobrevin 2 LI.  Ref: 10421869 J Comp Neurol, 1999
- Immunohistochemistry in combination with confocal laser microscopy showed the presence of synaptosomal associated protein of 25 kDa, syntaxin, cysteine string protein, synuclein,  **synapsin I** ,  **synapsin I**  II,  **synaptotagmin**  I,  **synaptotagmin**  I II, synaptophysin, and synaptobrevin 2 like immunoreactivity in VAChT containing C terminals.  Ref: 10421869 J Comp Neurol, 1999
- The effect of LTX on phosphorylation of synaptosomal proteins was studied including  **synapsin I** ,  **synaptotagmin** , and dynamin.  Ref: 8998284 Biokhimiia, 1996
- The presence of synaptic vesicle associated proteins such as synaptophysin,  **synaptotagmin**  and  **synapsin I**  in the growth cones of regenerating axons indicates the possibility that exocytotic fusion of vesicles with the surface axolemma supplies the membranous components for the extension of regenerating axons.  Ref: 8829147 Neurosci Res, 1996
- To ascertain the role of transmitter release from nerve terminals in promoting the ischemic neurodegeneration, we delivered antisense oligodeoxynucleotides ODNs to  **synaptotagmin**  I or  **synapsin I**  into the rat brain by using HVJ liposome gene transfer technique.  Ref: 12631464 Neurosci Res, 2003
- Antisense in vivo knockdown of  **synaptotagmin**  I and  **synapsin I**  by HVJ liposome mediated gene transfer modulates ischemic injury of hippocampus in opposing ways.  Ref: 12631464 Neurosci Res, 2003
- **Synaptotagmin**  I, vamp 2, and  **synapsin I**  proteins are synthesized at nearly constant rates as the neurons develop.  Ref: 9065497 J Neurosci, 1997
- BDNF also increased the levels of the vesicle proteins synaptophysin, synaptobrevin, and  **synaptotagmin** , without affecting the presynaptic membrane proteins syntaxin and SNAP 25, or the vesicle binding protein  **synapsin I** .  Ref: 11483592 J Biol Chem, 2001
